# Community Structure of Skipper Butterflies (Lepidoptera, Hesperiidae) along Elevational Gradients in Brazilian Atlantic Forest Reflects Vegetation Type Rather than Altitude

**DOI:** 10.1371/journal.pone.0108207

**Published:** 2014-10-01

**Authors:** Eduardo Carneiro, Olaf Hermann Hendrik Mielke, Mirna Martins Casagrande, Konrad Fiedler

**Affiliations:** 1 Laboratório de Estudos de Lepidoptera Neotropical, Zoology Department, UFPR. Curitiba, Paraná, Brasil; 2 Division of Tropical Ecology & Animal Biodiversity, University of Vienna, Vienna, Austria; University of Guelph, Canada

## Abstract

Species turnover across elevational gradients has matured into an important paradigm of community ecology. Here, we tested whether ecological and phylogenetic structure of skipper butterfly assemblages is more strongly structured according to altitude or vegetation type along three elevation gradients of moderate extent in Serra do Mar, Southern Brazil. Skippers were surveyed along three different mountain transects, and data on altitude and vegetation type of every collection site were recorded. NMDS ordination plots were used to assess community turnover and the influence of phylogenetic distance between species on apparent community patterns. Ordinations based on ecological similarity (Bray-Curtis index) were compared to those based on phylogenetic distance measures (MPD and MNTD) derived from a supertree. In the absence of a well-resolved phylogeny, various branch length transformation methods were applied together with four different null models, aiming to assess if results were confounded by low-resolution trees. Species composition as well as phylogenetic community structure of skipper butterflies were more prominently related to vegetation type instead of altitude per se. Phylogenetic distances reflected spatial community patterns less clearly than species composition, but revealed a more distinct fauna of monocot feeders associated with grassland habitats, implying that historical factors have played a fundamental role in shaping species composition across elevation gradients. Phylogenetic structure of community turned out to be a relevant additional tool which was even superior to identify faunal contrasts between forest and grassland habitats related to deep evolutionary splits. Since endemic skippers tend to occur in grassland habitats in the Serra do Mar, inclusion of phylogenetic diversity may also be important for conservation decisions.

## Introduction

Since the early observations by Forster [1] and von Humboldt [2], species turnover across altitudinal gradients has matured into an important paradigm of community ecology. High mountain chains are usually selected for analyses, mainly because their wide elevational ranges increase the power of detecting patterns in community structure (see [3], [4]). Geologically older mountain chains whose peaks are considerably lower as a consequence of long exposition to erosion forces have received less attention, since effects of elevation on species diversity or species composition might not be as evident. In such places, historical events may play an additional fundamental role in assemblage turnover in addition to altitude. More recently, the integration of phylogenetic structure into community ecology has yielded important insights as to how historical factors influence community structure [5].

The Serra do Mar is located near the south-eastern coast of Brazil and represents a good example of an old mountain chain in the southern hemisphere, where animal and plant communities might have been structured through both ecological and historical processes. The start of its rising processes has been dated at about 90 mya, soon after the splitting of Gondwana into its two biggest daughter continents, viz. South America and Africa [6]. Despite its moderate altitudinal amplitude today (peaks generally between 800–1500 m), distinct climatic and pedological shifts occur along these rather short elevational gradients, together with a high endemism rate in certain plant taxa such as Bromeliaceae, Orchidaceae and Gesneriaceae [6], [7].

Environmental dimensions change in a predictable manner with increasing altitude in the Serra do Mar. Mean annual temperature decreases by an average of 0.5–0.6 K per 100 m altitude, while soil depth decreases and wind intensity increases [8]. Correlated to those abiotic factors and modulated through superimposed effects of anthropogenic land-use, four different vegetation types can be observed on these mountains: Atlantic rain forest, early succession flora, cloud forest, and grasslands [7]. While Atlantic rain forest prevails in the lowlands (up to 1100 or 1400 m, depending on the mountain), grasslands are generally restricted to altitudes above 1300 m. These grasslands are relict vegetation ecosystems which had been far more widespread in south and southeast Brazil during dry cold periods of the late Quaternary [9]. Since then, this vegetation has become increasingly restricted to mountain tops, due to forest expansion, with many animal species related to this kind of ecosystem being included in red lists [10], [11]. Early successional flora is not only found after human interference, but also due to natural disturbance events such as forest fires or landslides [7], [8].

Communities of host-specific herbivores, whose life histories are intrinsically related to the vegetation of their habitats, may either respond directly to abiotic environmental gradients, or alternatively their species turnover may rather track biotic changes in vegetation types [12], [13]. The challenge remains to disentangle whether abiotic (climate, area size) or biotic conditions (vegetation) are more important as drivers of their community composition and species turnover along elevational gradients [14], [15], and how phylogenetic history contributes to understanding contrasting patterns between taxa [5], [16].

Various groups of nocturnal Lepidoptera have frequently served as targets to study elevational diversity patterns in the tropics [17]–[19], yet surprisingly few quantitative butterfly studies do exist from elevational gradients on tropical mountains [20], [21]. Especially, the few studies addressing phylogenetic structure in butterfly communities along elevational gradients are extra-tropical [22], [23], where faunas are distinctly less diverse (but see [24] for a recent example on a rich Neotropical moth assemblage). Skipper butterflies (Lepidoptera, Hesperioidea) might also have the potential to address elevational patterns and underlying processes, but thus far no specific case study on this somehow elusive family exists from mountain ranges anywhere in the tropics.

Recent studies revealed contrasting patterns of phylogenetic community structure along environmental gradients, i.e. different assemblages respond to environmental or biotic factors depending on their specific requirements, evolutionary history and biogeography [25]–[28]. Skippers might have particular potential to reveal how vegetation types and altitude influence phylogenetic composition along altitudinal gradients, since major skipper lineages are conservative and contrasting in relation to their larval food plant affiliations [29]. While Hesperiinae larvae feed exclusively on monocotyledonous plants, Pyrginae larvae are bound to various families of dicotyledonous plants, mainly Fabaceae, Malvaceae and Rutaceae [29]–[31]. In addition, skippers are the only group of butterflies commonly observed throughout all sections of altitudinal gradients in south Brazil (pers. observ.), and they comprise far larger numbers of grassland endemics when compared to Papilionoidea families in the region [32].

The present study aimed to elucidate the structure of Hesperiidae assemblages along altitudinal gradients of moderate extension, using ecological and phylogenetic measures of community similarity, on three different mountains, in relation to different vegetation types. Specifically, the following hypotheses were tested: 1. Assemblages of skippers are structured according to vegetation type as well as altitude; 2. Phylogenetic similarity is more informative for community responses to altitude and vegetation than ecological similarity (e.g. species composition); 3. Vegetation has a stronger influence on skipper assemblages than altitude; 4. Subfamilies of skippers show discordant community patterns because they differ in life-history traits governing their habitat preferences.

## Methods

### Study location and sampling methods

Three mountains located in the Serra do Mar of Paraná state, Brazil were sampled in this study: Anhangava (25°23′30″S; 49°00′15″W), Araçatuba (25°54′07″S; 48°59′37″W) and Caratuva mountain (25°13′30″S; 48°51′40″W). The three locations sampled are embedded in protected areas regulated by the state environmental agency (IAP/PR). For location details see [33]. Capture of specimens and their transport to the laboratory for subsequent identification were permitted by licences n° 59.08 (IAP/PR) and 14.595-1 (IBAMA/Sisbio). Hesperiidae specimens were captured with insect nets during up- and down-walking of transects. Each transect was walked 11 times from 2009 to 2011, between 9∶00 and 16∶00 h. All recorded specimens were immediately labeled according to the elevation (measured to the nearest 10 m using a Garmin 60Cx GPS device) and vegetation type of their sampling locality. In all, 1578 records of 155 species make up the data on which all analyses are based. No endangered species were recorded in this study.

On the mountains in Serra do Mar up to four different vegetation types are present above 900 m: montane forest, cloud forest, early successional vegetation, and grassland. Montane forest refers to a well-developed, tall grown (up to 10 m), vertically stratified Atlantic Rainforest located throughout the slopes of Serra do Mar. Cloud forest stands are more dense, lower in growth (tree height from 3–7 m) with less well defined strata. Successional vegetation and grasslands both lack a canopy stratum. Succession vegetation is dominated by bracken fern *Pteridium aquilinum* (Dennstaedtiaceae), and it is often located where fire or human impact have occurred recently [8]. Grassland sites present a varied floral composition, dominated by several species of Poaceae and Cyperaceae, but occasionally with scattered low trees and shrubs [7].

Although these vegetation types are in part related to altitude (e.g. grasslands are located on mountain tops), their ranges also vary according to a mountain’s relief, soil depth, or biogeographical history [7]. This peculiarity allowed us to examine whether vegetation or altitude *per se* plays the more important role on community differentiation. Therefore, skipper samples were delimited and analyzed in two different ways: first, the whole altitudinal range on each mountain was divided into belts of 100 m elevational extent, without considering changes of vegetation types within; second, samples were again delimited by altitude, but in addition the prevalent vegetation type was superimposed to delimit sample sites (Table 1). As a consequence, these latter operational units were not equally sized according to elevational bands, but varied from 50 to 75 m extension each. Because only two specimens of skippers were collected in cloud forest, this vegetation type was excluded from all analyses.

**Table 1:** Two different sets of sample unit delimitations used to analyze skipper assemblages in the Serra do Mar (Brazil)^1^.

Samples 1 (m)	Mountain	Veg. Type	Samples 2 (m)	Mountain	Veg. Type	Altitude Class
1000–1100	Anhangava	FOR	998–1060	Anhangava	FOR	low
1100–1200	Anhangava	FOR+ESV	1061–1122	Anhangava	FOR	low
1200–1300	Anhangava	ESV+GRA	1123–1206	Anhangava	ESV	medium
1300–1400	Anhangava	GRA	1207–1289	Anhangava	ESV	medium
1400–1500	Anhangava	GRA	1290–1364	Anhangava	GRA	medium*
900–1000	Araçatuba	FOR+ESV	1365–1440	Anhangava	GRA	high
1000–1100	Araçatuba	ESV	912–938	Araçatuba	FOR	low
1100–1200	Araçatuba	GRA	939–1019	Araçatuba	ESV	low
1200–1300	Araçatuba	GRA	1020–1099	Araçatuba	ESV	low
1300–1400	Araçatuba	GRA	1100–1175	Araçatuba	GRA	low
1400–1500	Araçatuba	GRA	1176–1250	Araçatuba	GRA	medium
1500–1600	Araçatuba	GRA	1251–1325	Araçatuba	GRA	medium
1600–1700	Araçatuba	GRA	1326–1400	Araçatuba	GRA	medium
900–1000	Caratuva	FOR	1401–1475	Araçatuba	GRA	high
1000–1100	Caratuva	FOR+ESV	1476–1550	Araçatuba	GRA	high*
1100–1200	Caratuva	ESV	1551–1625	Araçatuba	GRA	high
1200–1300	Caratuva	ESV	1625–1682	Araçatuba	GRA	high
1300–1400	Caratuva	FOR	980–1031	Caratuva	FOR	low
1400–1500	Caratuva	FOR+GRA	1032–1083	Caratuva	FOR	low
1800–1900	Caratuva	GRA	1084–1158	Caratuva	ESV	low
			1159–1233	Caratuva	ESV	medium
			1234–1306	Caratuva	ESV	medium
			1307–1362	Caratuva	FOR	medium
			1363–1418	Caratuva	FOR	medium
			1419–1488	Caratuva	GRA	high
			1800–1860	Caratuva	GRA	high*

^1^Samples 1: delimited only by altitude; Samples 2: delimited by vegetation type and altitude. Each location is assigned to mountains, elevational belts and vegetation types. Note that the delimitation by altitude plus vegetation increases the number of sample units. Abbreviations: FOR: forest; ESV: early successional vegetation; GRA: grassland.

### Ecological and phylogenetic community structure analyses

NMDS ordinations were used to search for both ecological and phylogenetic structure in skipper assemblages along elevational gradients. This methodology enables the recognition of spatial gradients across communities through comparisons of pairwise similarities, or distances, between all samples [34]. Brehm & Fiedler [35] evaluated different ordination methods for identifying elevational gradients with incompletely sampled communities and concluded that different techniques performed quite similarly. Furthermore, NMDS has the advantage of fixing *a priori* the number of dimensions to be considered for analysis, and to be grounded on rank statistics which renders this ordination method very robust [36], [37].

In a first series of NMDS explorations, based on Bray-Curtis matrix similarities, it was assessed whether inclusion, or exclusion, of hilltopping species (i.e. where adult butterflies aggregate at mountain tops for mate location [38]), or the segregation of samples only by altitude (100 m belts), or by altitude plus vegetation type (50–75 m belts), would affect ordination patterns, as already shown for species richness patterns [33]. Since both these factors indeed influenced the ordination of assemblages (Fig. S1), in the subsequent main series of analyses all hilltopping species were omitted and sample sites were classified according to elevation plus vegetation type. Species that exhibit hilltopping behavior are listed in Carneiro et al. [33]. Because hilltopping species were quite numerous, two samples, one from the 1800–1900 m band on Caratuva and the 1476–1550 m band on Araçatuba, became too small and therefore had to be excluded altogether. As shown before [33], neither vegetation type nor altitude influenced the efficiency of sampling skippers. Therefore, differences in sample coverage are not expected to affect our results.

The Bray-Curtis similarity index was used to measure ecological similarities of assemblages. Complementarily, the incidence-based Chao-Soerensen index [39] was also calculated, but this did not yield any deviant patters (data not shown). Additionally, two indexes were calculated to assess phylogenetic distances between assemblage samples: the Mean Pairwise Distance (MPD) and the Mean Nearest Neighbor Distance (MNTD) [5]. Both indices were compared because they provide different perspectives of phylogenetic similarities, as an overall pattern of relatedness (MPD) or as how closely related species can be (MNTD) [40]. Additionally, MNTD is more affected by changes at the terminal branches of a phylogeny, whereas MPD is more sensitive to changes at the basis of a phylogeny [40].

To calculate measurements of phylogenetic structure, a phylogenetic hypothesis of the sampled taxa is obviously required. Skipper phylogeny is still very imperfectly resolved [29], [41]. Based on the latest phylogenetic approach [29], a tentative community supertree was constructed. Topology of high rank taxa was recovered after [29]. Groups (G) and subgroups (SG) stated by Evans [42] were maintained only when they did not conflict with the current tree topology [29]. Species were clumped according to their respective genera (Fig. S2). When genera or species sampled in our study had not been included in the phylogenetic analyses, they were replaced by their closest tribal affiliates, using expert taxonomical arrangement as best possible surrogate of phylogenetic support [40], [43]. Because branch length estimates were not available, and even node ages and fossil calibrations are still largely questioned in butterfly systematics [44], equal branch lengths were arbitrarily assigned to the tree topology (which is the same approach as the nodal distance method; [40]). We then compared these results with branch lengths using Grafen’s Rho transformation (r = 0.5) [45] (Fig. S2). Additional branch length transformation methods were also calculated (e.g. Pagel’s and Nee’s methods: [46], [47]), but results did not systematically differ from those presented below (data not shown).

To ascertain that phylogenetic structure of communities obtained from the supertree and branch transformation techniques were different from what would be expected at random, we correlated the empirical phylogenetic distance matrices with matrices produced by four distinct null models (means from 1000 randomizations) through Spearman matrix rank correlation tests (procedure RELATE in PRIMER: [48]). These null models included the shuffling of species labels across the phylogeny (null 0), randomization of species from the sample pool (null 1), randomization of species from the phylogeny pool (null 2), or by swapping versions of sample/species matrix (see [49], [50] for details). The choice of an adequate null model is relevant, since their different assumptions might lead to contrasting results [51]. Because our phylogenetic trees do not contain branch length information based on genetic differences, we opted for challenging the results obtained through all these null models and then interpret results.

To test whether taxonomic scale may be informative to community similarities between different altitude and vegetation types, the analyses was first performed for the whole family Hesperiidae, and then separately for communities composed only of Pyrginae+Pyrrhopyginae species (i.e. dicot feeders, hereafter called Pyrginae); or only of Hesperiinae (i.e. monocot feeders). *Urbanus teleus* (Hübner, 1821) was included in the Hesperiinae analyses, since this species has confirmed host-plant records only in Poaceae [29], disregarding a probably misleading record from Fabaceae [31]. Pyrginae and Hesperiinae similarity matrices were thereafter compared across the same common sampling sites by Spearman matrix rank correlation, aiming to test whether taxa with such contrasting hostplant associations differ in their ecological and phylogenetic similarity patterns in response to elevation and vegetation change. Vegetation and altitude were classified as factors and tested in two-way ANOSIM analyses, where R statistics values served as a measure of effect size. Vegetation was divided into forest, early successional vegetation, and grassland, while the elevational categories included in this analyses were ‘low’ (900 m–1150 m), ‘medium’ (1150 m–1400 m), and ‘high’ (1400 m–1650 m; see Table 1).

Bray-Curtis similarity indexes, NMDS ordinations, RELATE tests and two-way ANOSIM were calculated with PRIMER 6.1.13 [48]. Phylogenetic trees were drawn and branch lengths put in and transformed using Mesquite 2.72 [52], including the PDAP Package [53]. Phylogenetic community distances were calculated using COMDIST and COMDISTNT functions available in PHYLOCOM 4.2 [54]. To avoid spurious significance resulting from multiple tests, a “False Discovery Rate” approach [55] was taken [56]. All test results passing adjusted criteria were assigned with an asterisk (*).

## Results

### Ecological and phylogenetic community structure at family level

**Figure 1:**
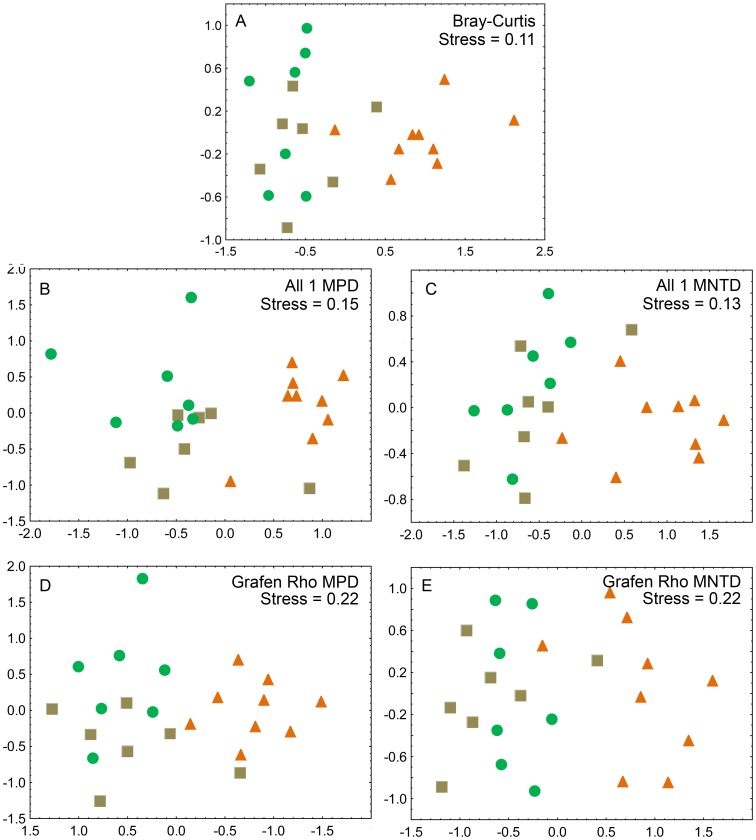
NMDS ordination plots of Hesperiidae assemblages along elevational gradients in Serra do Mar, Brazil. Ordination patterns were assessed based on Bray-Curtis similarities of species lists (A), and compared to two phylogenetic distance indexes (MPD and MNTD) using equal branch lengths (All 1) and Grafen’s Rho transformation method (charts B–E). Samples are scored according to altitude and vegetation types. Assemblages are basically ordered along the first axis from low (left) to high elevations (right). Symbols: green circles (forest), brown squares (early successional vegetation), orange triangles (grassland). Stress values indicate goodness of fit of two-dimensional representations to the underlying distance matrices.

The total skipper fauna collected along all transects comprised of 1578 specimens representing 155 species. Spatial resolution revealed by NMDS ordinations uncovered a nearly even gradient-like pattern mainly represented by the first ordination axis, along which communities were ordered from high to low altitudes (Fig. 1a, ‘Bray-Curtis’ in Table 2). However, assemblages from grassland sites (from elevations between 1100–1650 m elevation) became segregated from all others, whereas skipper assemblages from forest and early successional vegetation tended to be spatially unordered along both axes. Therefore, an elevational gradient was not evident when looking exclusively to points within each vegetation type, indicating that elevation is secondary to vegetation type in shaping species composition.

**Table 2:** Spearman rank correlation coefficients *r* (plus associated *p*-values) between altitude of sample sites and the site scores along the two ordination axes extracted from NMDS ordinations^1^.

	NMDS Axis 1		NMDS Axis 2	
	*r*	*p*	*r*	*p*
Bray-Curtis	**0.67**	**0.001**	0.31	0.148
MPD All1	**0.54**	**0.008**	0.07	0.758
MPD Grafen’s Rho	**0.53**	**0.009**	0.06	0.792
MNTD All1	**0.54**	**0.008**	**0.60**	**0.002**
MNTD Grafen’s Rho	**0.49**	**0.019**	**0.49**	**0.017**

^1^Different sets of Hesperiidae assemblages were considered with different measures of species or phylogenetic composition, sampled along altitudinal gradients in Serra do Mar, Paraná, Brazil. ‘All 1' refers to equal branch lengths assigned to the tree topology while ‘Grafen’s Rho’ refers to Grafen’s branch length transformation method [45]. Correlations that remain significant after applying a table-wide false discovery rate approach are printed in bold face.

When skipper assemblages were ordinated by their phylogenetic distances in the MPD model, spatial patterns were remarkably similar, and NMDS representations achieved almost equal goodness-of-fit (Figs. 1b–e). Application of various branch length transformations did not alter the general pattern observed in NMDS ordinations, but two-dimensional representations had poorer fit (i.e. higher stress values and lower correlations of ordination axis scores with altitude) than if assuming equal branch lengths set to unity. Ordinations based on MPD and MNTD measures, respectively, showed similar patterns with regard to group clustering. Axis 2 significantly correlated with altitude in ordinations based on MNTD, but not for those using MPD (Table 2). Spearman rank correlations between the ecological Bray-Curtis similarity matrix and the four phylogeny-based distance matrices (MPD and MNTD with two branch-length options each) were always highly significant (*p*≤0.001), but MNTD index showed higher coefficient values with Bray-Curtis ecological index (0.84 to 0.87), compared to MPD (0.50 to 0.60) (Table 3). Strikingly, phylogenetic indexes showed the lowest coefficient values of correlation between each other (0.35 to 0.52).

**Table 3:** Pairwise Spearman rank correlation coefficients *r* (plus associated *p*-values) for the similarity matrices extracted by ecological and phylogenetic metrics^1^.

	Bray-Curtis		All 1 MPD		All 1 MNTD		Grafen Rho MPD	
Bray-Curtis	–	–						
All 1 MPD	**0.509**	**<0.001**	**–**	**–**				
All 1 MNTD	**0.849**	**<0.001**	**0.397**	**0.001**	**–**	**–**		
Grafen Rho MPD	**0.608**	**<0.001**	**0.787**	**0.001**	**0.498**	**<0.001**	**–**	**–**
Grafen Rho MNTD	**0.877**	**<0.001**	**0.357**	**0.001**	**0.957**	**<0.001**	**0.522**	**<0.001**

^1^Ecological ordination was measured with Bray-Curtis similarity matrix, while four phylogeny-based distance matrices (MPD and MNTD) were calculated using two branch-length options each. ‘All 1’ refers to equal branch lengths assigned to the tree topology while ‘Grafen’s Rho’ refers to Grafen’s Rho branch length transformation method [45]. Correlations that remain significant after applying a table-wide false discovery rate approach are printed in bold face.

Differences between MPD and MNTD distance measures were particularly obvious with regard to null-model tests (Table 4). The MNTD matrix correlated strongly with random matrices created under any of the four null models compared. In contrast, correlations between the MPD matrix and null models were only significant (and much weaker so) when all branch lengths were assigned to one, i.e. in null models 0 and 3 (shuffling of phylogenetic terminal labels and swapping species between samples, respectively). Therefore, MNTD results cannot be reliably interpreted as relating to ecological conditions and we restricted further considerations to MPD measures.

**Table 4:** Spearman matrix rank correlation coefficients *r* (999 permutations) between phylogenetic distance matrices MPD (mean pairwise distance) and MNTD (mean nearest neighbor distance and four types of null models: 0–3 [49], [50] ^1^.

	Model 0		Model 1		Model 2		Model 3	
	*r*	*p*	*r*	*p*	*r*	*p*	*r*	*p*
**Hesperiidae**								
**MPD**								
All 1	**0.41**	**0.001**	0.05	0.725	0.02	0.580	0.22	0.030
Grafen Rho	0.06	0.239	0.05	0.303	0.05	0.300	0.04	0.648
**MNTD**								
All 1	**0.83**	**0.001**	**0.48**	**0.001**	**0.48**	**0.001**	**0.50**	**0.001**
Grafen Rho	**0.17**	**0.013**	**0.27**	**0.003**	**0.27**	**0.001**	**0.37**	**0.001**
**Hesperiinae**								
**MPD**								
All 1	**0.60**	**0.001**	0.00	0.524	0.06	0.782	−0.23	0.996
Grafen Rho	0.02	0.376	0.05	0.248	−0.02	0.671	−0.09	0.935
**MNTD**								
All 1	**0.69**	**0.001**	**0.33**	**0.001**	**0.33**	**0.001**	**0.33**	**0.001**
Grafen Rho	**0.28**	**0.001**	**0.18**	**0.003**	**0.28**	**0.001**	**0.20**	**0.002**
**Pyrginae**								
**MPD**								
All 1	**0.28**	**0.013**	0.11	0.209	−0.08	0.753	**0.36**	**0.023**
Grafen Rho	0.18	0.081	−0.32	0.973	−0.05	0.658	−0.07	0.665
**MNTD**								
All 1	**0.44**	**0.001**	**0.24**	**0.018**	0.18	0.097	**0.31**	**0.001**
Grafen Rho	0.19	0.091	0.18	0.092	0.14	0.153	0.17	0.097

^1^Two different branch length transformation methods were applied with the mean of 999 randomly generated matrices, according to four null models assumptions (see Methods section). ‘All 1’: equal branch lengths assigned to the tree topology; ‘Grafen’s Rho’: Grafen’s branch length transformation method [45]. Significant correlations here indicate that observed sample matrices are not substantially different from random expectations. Null model tests were applied to the three taxa Hesperiidae, Hesperiinae and Pyrginae. Correlations that remain significant after applying a table-wide false discovery rate approach are printed in bold face.

### Effects of vegetation, altitude and taxon scale: Pyrginae vs. Hesperiinae

**Figure 2:**
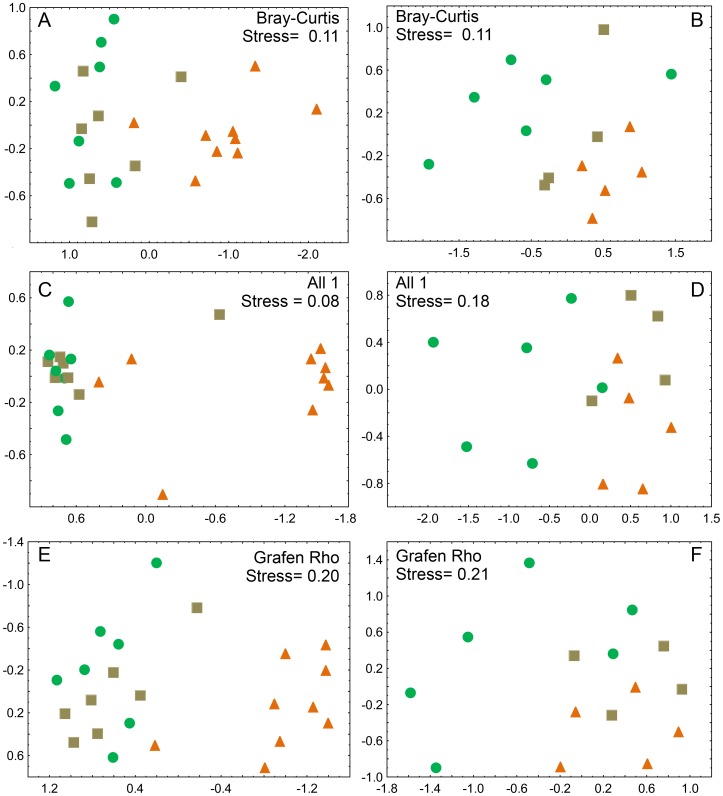
NMDS ordination plots of Hesperiinae (left panels, with monocot-feeding larvae) and Pyrginae (right panels, with larvae feeding on various dicot families) assemblages along elevational gradients in Serra do Mar, Paraná, Brazil. Ordinations are based on ecological (Bray-Curtis: A, B) and phylogenetic (MPD) similarity indexes. Two branch length transformations were applied to obtain MPD: ‘All 1’ (all branches set to unity: C, D) and Grafen’s Rho model (E, F). Samples are partitioned according to altitude and vegetation types. Symbols: green circles (forest), brown squares (early successional vegetation), orange triangles (grassland). Stress values indicate goodness of fit of two-dimensional representations to the underlying distance matrices.

When analyzed separately, the two subfamilies Hesperiinae and Pyrginae revealed important differences in their patterns of assemblage similarities (Fig. 2). The NMDS ordination of Hesperiinae rendered a similar configuration as the entire family (Fig. 2a). Grassland skipper assemblages were distinctly set apart, with no clear differentiation between communities associated with forest and early successional vegetation. Pyrginae assemblages, in contrast, revealed a more distinct grouping of forest sites, as opposed to non-forested grassland and early successional sites (Fig. 2b). Ordinations based on phylogenetic distances were qualitatively similar, but tended to show less segregation between vegetation types, especially for the Pyrginae (Figs. 2c–f). These observations are supported by ANOSIM tests. Vegetation type was the main factor responsible for governing Hesperiidae and Hesperiinae assemblages, rather than altitude *per se* (Table 5). *R* values for comparisons based on phylogenetic distance measures were generally lower than those for comparisons of species composition. This was particularly pronounced in the subfamily Pyrginae.

**Table 5:** Two-way ANOSIM results (*R* statistics and associated *p*-values), evaluating the effects of vegetation type and altitude on ecological similarities (Bray-Curtis) and phylogenetic distances (MPD) of skipper assemblages^1^.

	Vegetation		Altitude	
	R^2^	p	R^2^	p
**Hesperiidae**				
Bray-Curtis	**0.47**	**0.004**	**0.10**	**0.001**
All1	0.19	0.13	0.13	0.81
Grafen Rho	**0.35**	**0.006**	0.01	0.5
**Hesperiinae**				
Bray-Curtis	**0.46**	**0.005**	0.05	0.328
All1	**0.49**	**0.004**	0.13	0.18
Grafen Rho	**0.39**	**0.013**	0.08	0.279
**Pyrginae**				
Bray-Curtis	**0.64**	**0.003**	0.76	0.03
All1	**0.08**	**0.298**	0.39	0.852
Grafen Rho	0.06	0.507	0.40	0.856

^1^Analyses were performed at three different taxonomic levels (entire family Hesperiidae, and two major subfamilies), and for two different branch length transformation methods: ‘All 1’: equal branch lengths assigned to the tree topology; ‘Grafen’s Rho’: Grafen’s branch length transformation method [45]. Results printed in bold remained significant after applying a false discovery rate approach.

## Discussion

### Ecological and phylogenetic community structure

Hesperiidae assemblages differed greatly in their species composition across elevational transects in Serra do Mar, even though sampling occurred over a moderate altitudinal range of 500–700 m extension. Although some herbivorous insect taxa are especially sensitive to abiotic gradients related to altitude [57], [58], vegetation may exert more direct and distinct effects on species turnover. This was clearly the case with skipper assemblages in our study. Species turnover along elevational gradients has regularly been demonstrated to occur in many groups of plants and animals (for tropical butterflies and moths, e.g. [18], [21]). Yet those studies often required far more extensive altitudinal ranges to uncover species turnover patterns.

In unconstrained ordinations, ecological species composition patterns were strikingly similar to those inferred from phylogenetic distances such as MPD and MNTD. In contrast to the number of studies investigating ecological species turnover along elevational gradients, fewer studies thus far attempted to address phylogenetic turnover in community compositions. This is due to the lack of robust phylogenetic hypotheses for most invertebrate groups, especially in tropical biota. In our case, the finding that MNTD distance patterns were not different from random expectations, opposite to MPD, might reflect the inaccuracy of our phylogenetic tree, in which terminal branches are less well established (presence of many polytomies) than is the resolution among higher level groups [29], [41]. Skipper phylogeny has been addressed only recently, and molecular information from many genera, especially of Neotropical origin, is still completely lacking. Nevertheless, MPD data allowed us to reveal almost the same spatial pattern as abundance-weighted species turnover. This corroborates that phylogenetic distance information can be relevant also in groups with substantial uncertainty about their phylogenetic relationships [40], [59].

Even though different null models, as expected, showed divergent results when applied to skippers [49], [51], a non-random general pattern with regard to phylogenetic community distances was clearly observed. In general, various branch length transformation methods did not massively affect the outcome of ordination analyses, but the assumption of equal branch lengths reduced the information content to levels of random relationships between samples. Therefore, trees without genetically founded branch length estimates can still furnish consistent results [40], once different indexes are used and compared to a variety of null models to verify *a priori* whether phylogenetic inaccuracy may have obscured ecologically relevant aspects of phylogenetic community structure.

### Effects of vegetation, altitude and taxon scale: Pyrginae vs. Hesperiinae

While numerous studies have elucidated how elevational gradients influence community similarities, only a few specifically addressed the relevance of vegetation types in relation to merely abiotic gradients on animal assemblages [12], [60]–[62]. This integration, however, is essential, since high altitude habitats often represent different vegetation types which not necessarily are concordant to climatic change with elevation alone [14]. In cases where vegetational and abiotic dimensions were related to butterfly or moth assemblages, altitude (and climate) usually emerged as the single best predictor of faunal composition or richness instead of habitat type [12], [60], [62]. Accordingly, vegetation type at most emerged as modulating patterns of elevational faunal change [61], [63], [64].

In contrast, vegetation type played the major role with regard to community differentiation in skipper butterflies of Serra do Mar, instead of altitude *per se*. Along elevational transects of moderate extent, factors beyond mere laps rate may gain higher relative importance, as temperature differences are less prominent than on high mountain ranges like the Andes or Himalaya [4]. Accordingly, the presence of distinct vegetation types should attain higher weight in elevation gradients studies, addressing not only a novel ecological constraint (e.g. humidity and solar incidence are clearly contrasting between grasslands and forests), but also evolutionary dimensions, such as insect-plant interactions.

Life history traits are particularly related to the diversification of Hesperiidae. The two major subfamilies are rather conservative in larval food plant affiliations with either monocot or dicot plant families [29]. Hesperiinae (exclusive monocot feeders) are a more recent lineage than all other dicot feeders, with almost twice the global species richness of Pyrginae [29]. Hence diversification rate in this group must be distinctly higher, in contrast to the relatively low diversification rate estimated for the entire family [65]. Another unrelated group of monocot feeding butterflies (viz. Satyrinae) displays a similar pattern of speciation, and the expansion of grassland habitats around the world has been linked to this “explosive diversification” [66]. The high number of Hesperiinae species endemic to grasslands ecosystems in south Brazil reinforces this statement.

Hesperiinae assemblages of grassland sites were more distinctly clustered when analyses were based on phylogenetic distances rather than abundance-weighted species lists. This may hint towards a historical relationship with grassland ecosystems through evolutionary time. In other words, skipper species in this highly distinct vegetation type represent lineages with unique evolutionary history [16]. Consequently, environmental filtering caused by altitudinal climate shifts seems to play only a secondary role in structuring those assemblages.

### Implications for Conservation

Conservation of natural grasslands ecosystems is of transcontinental concern [67]. Atlantic forests are biodiversity hot-spots not only because of high species richness of organisms, but also because of high numbers of endemics [68]. Because mountain grassland patches in the Serra do Mar are inserted amongst this forest landscape, endemic organisms of grasslands are routinely counted to this ecosystem, especially because fine scale distribution of grassland patches is hardly represented in distribution maps. Grassland ecosystems embedded in Atlantic Forest are phylogenetically linked to unique skipper assemblages, in analogy to high-altitude grassland butterflies in the European Alps [23]. The reduction of grassland ecosystems in Atlantic Forest is historically related to climate fluctuations, but nowadays these ecosystems are also threatened by occasional anthropogenic fires, invasive exotic grasses and extensive tourism [69]. The present findings highlight the need of conserving these high altitude habitats also from the perspective of a unique skipper butterfly fauna. Although difficult to identify, Neotropical skippers comprise rich assemblages (compared for example to frugivorous butterflies), and are suitable sensitive biological indicators in Atlantic Forest [68]. Hence, further taxonomic and ecological studies into this family are desired, since for most skipper species we still lack information on hostplant associations, phylogenetic relationships, and geographical distribution records. Even, quite a number of unknown species remain to be described.

## Supporting Information

Figure S1 Explorative NMDS ordination plots of Hesperiidae assemblages along elevational gradients in Serra do Mar, Brazil. Ordination patterns were first assessed based on Bray-Curtis similarities, with samples partitioned into 100 m altitudinal bands (a), into 100 m altitudinal bands, but excluding hilltopping species (b), and partitioned according to altitude and vegetation types (excluding hilltopping species) c). Arrows indicate mountain summit samples.
(DOCX)

Figure S2 Phylogenetic relationships of Hesperiidae (Insecta, Lepidoptera) species recorded in Serra do Mar, Paraná, Brazil. Topology of high rank taxa was recovered after Warren et al. [29]. Groups (G) and subgroups (SG) stated by Evans [42] were maintained only when not conflicting with the topology published in Warren et al. [29]. Species were clumped according to its respectively genera. As no branch lengths are still available for skipper phylogeny, equal branch lengths (above) and Grafen’s Rho transformation (below) were arbitrarily assigned to quantify phylogenetic differences between species.
(DOCX)
