# Supplementary material for: Community Structure of Skipper Butterflies (Lepidoptera, Hesperiidae) along Elevational Gradients in Brazilian Atlantic Forest Reflects Vegetation Type Rather than Altitude
Source: PLoS One. 2014 Oct 1;9(10):e108207. doi: 10.1371/journal.pone.0108207 (PMC4182717; doi:10.1371/journal.pone.0108207)
Supplement: Figure S1 — Explorative NMDS ordination plots of Hesperiidae assemblages along elevational gradients in Serra do Mar, Brazil. Ordination patterns were first assessed based on Bray-Curtis similarities, with samples partitioned into 100 m altitudinal bands (a), into 100 m altitudinal bands, but excluding hilltopping species (b), and partitioned according to altitude and vegetation types (excluding hilltopping species) c). Arrows indicate mountain summit samples. (DOCX) [file pone.0108207.s001.docx]

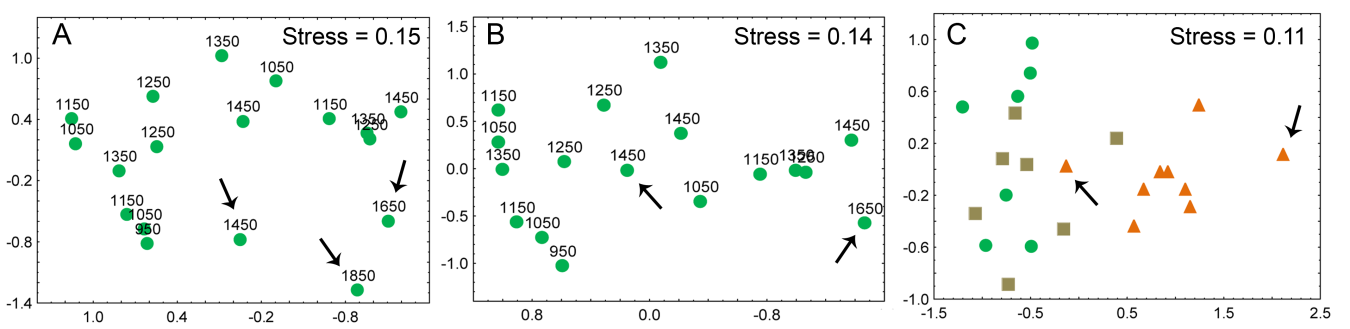


Figure S1: Preliminary NMDS ordination plots of Hesperiidae assemblages along elevational gradients in Serra do Mar, Brazil. Ordination patterns were first assessed based on Bray-Curtis similarities, with samples partitioned into 100m altitudinal bands (A), into 100m altitudinal bands, but excluding hilltopping species (B), and partitioned according to altitude and vegetation types (excluding hilltopping species) (C). Triangles represent community samples in grasslands, squares in early successional vegetation and circles in forests. All ordinations basically reveal an elevational gradient along the first axis from low (left) to high (right) altitudes. Arrows indicate summit samples.
