# Supplementary material for: Community Structure of Skipper Butterflies (Lepidoptera, Hesperiidae) along Elevational Gradients in Brazilian Atlantic Forest Reflects Vegetation Type Rather than Altitude
Source: PLoS One. 2014 Oct 1;9(10):e108207. doi: 10.1371/journal.pone.0108207 (PMC4182717; doi:10.1371/journal.pone.0108207)
Supplement: Figure S2 — Phylogenetic relationships of Hesperiidae (Insecta, Lepidoptera) species recorded in Serra do Mar, Paraná, Brazil. Topology of high rank taxa was recovered after Warren et al. [29]. Groups (G) and subgroups (SG) stated by Evans [42] were maintained only when not conflicting with the topology published in Warren et al. [29]. Species were clumped according to its respectively genera. As no branch lengths are still available for skipper phylogeny, equal branch lengths (above) and Grafen’s Rho transformation (below) were arbitrarily assigned to quantify phylogenetic differences between species. (DOCX) [file pone.0108207.s002.docx]

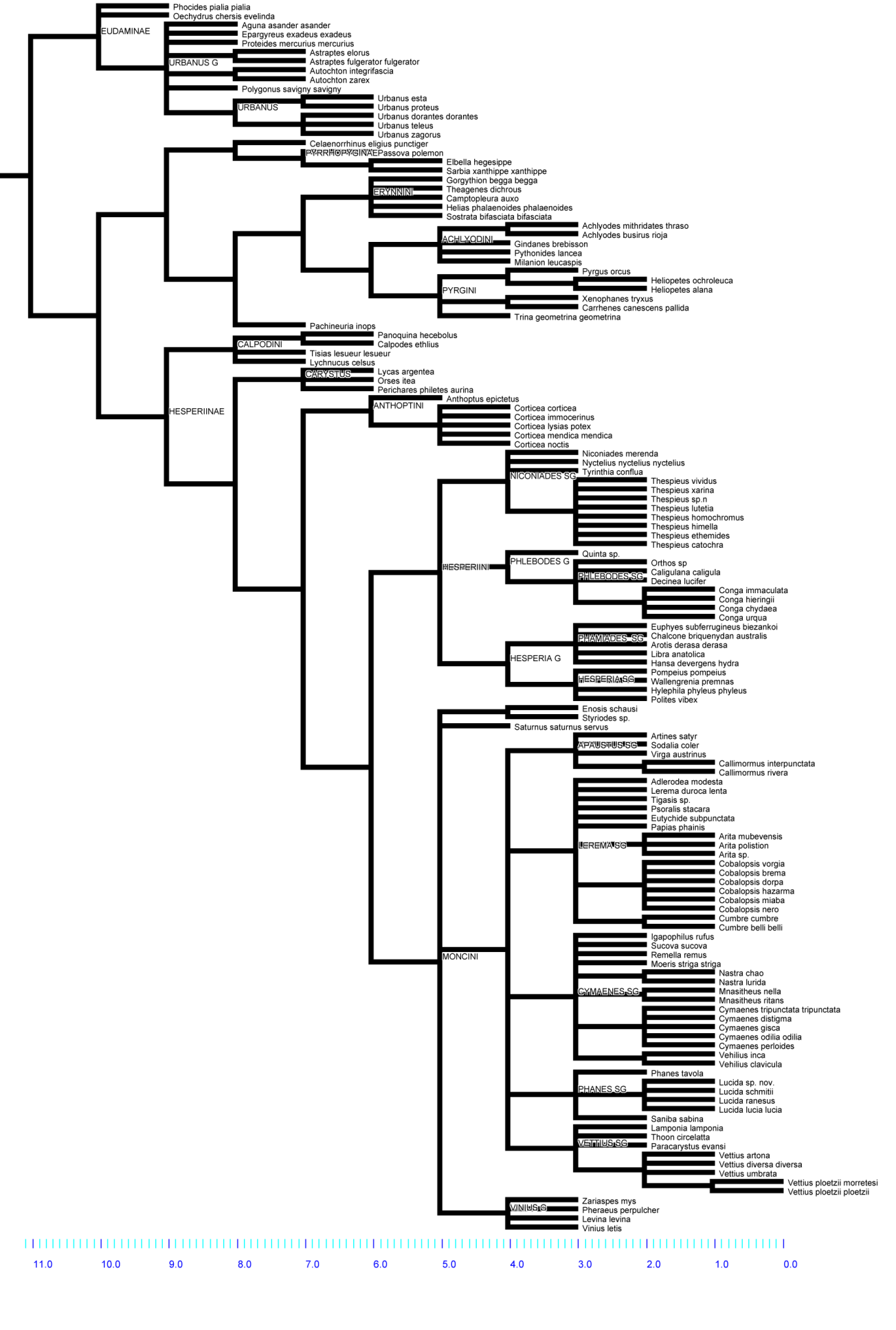

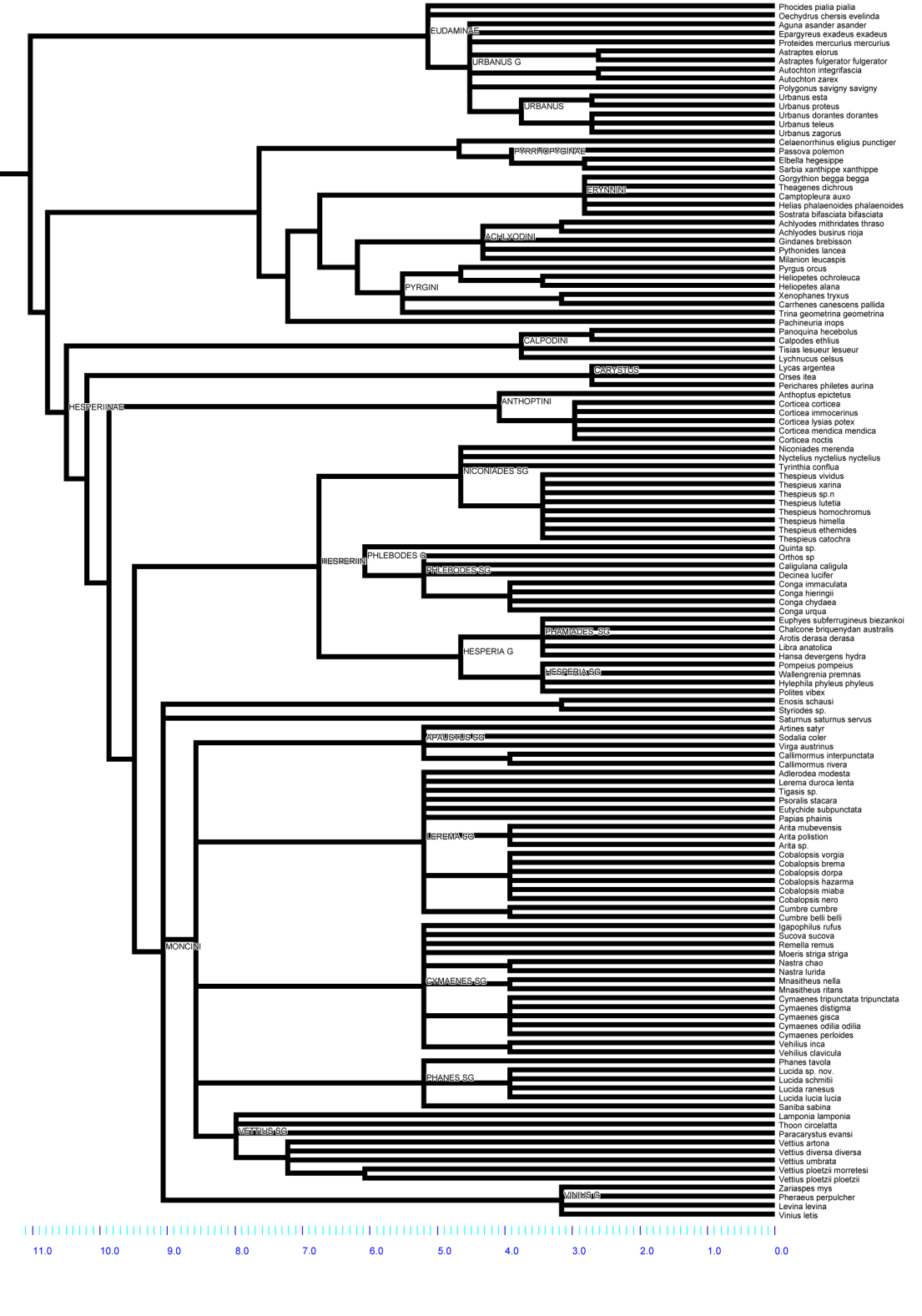


Figure S2: Phylogenetic relationships of Hesperiidae (Insecta, Lepidoptera) species recorded in Serra do Mar, Paraná, Brazil. Topology of high rank taxa was extracted from Warren et al. (2009). Groups (G) and subgroups (SG), as erected by Evans (1955) through traditional morphology-based systematics, were maintained only when not conflicting with the topology in Warren et al. (2009). Species were clumped according to their respective genera. As no branch length data are still available for skipper phylogeny, equal branch lengths (above) and Grafen's Rho transformation (below) were arbitrarily assigned to quantify phylogenetic differences between species.
